# Potential bias in testing for hyperprolactinemia and pituitary tumors in risperidone-treated patients: a claims-based study

**DOI:** 10.1186/1744-859X-8-5

**Published:** 2009-02-11

**Authors:** Frank D Gianfrancesco, Gahan Pandina, Ramy Mahmoud, Jasmanda Wu, Ruey H Wang

**Affiliations:** 1 HECON Associates Inc., 9833 Whetstone Drive, Montgomery Village, MD 20886, USA; 2 Johnson & Johnson Pharmaceutical Research and Development, 1125 Trenton-Harbourton Road, Titusville, NJ 08560, USA; 3 Ethicon, Inc. (a Johnson & Johnson Company), PO Box 151, Route 22 West, Somerville, NJ 08876-0151, USA; 4 Ortho-McNeil Janssen Scientific Affairs, LLC, 1125 Trenton-Harbourton Road, Titusville, NJ 08560, USA

## Abstract

**Background:**

A reporting association of risperidone with pituitary tumors has been observed. Because such tumors are highly prevalent, there may be other reasons why they were revealed in association with risperidone treatment. We assessed two potential explanations: disproportionately more prolactin assessment and head/brain imaging in risperidone-treated patients vs patients treated with other antipsychotics.

**Methods:**

Treatment episodes with risperidone, clozapine, olanzapine, quetiapine, ziprasidone, aripiprazole, haloperidol, perphenazine and 'other typical' antipsychotics were identified in two databases (large commercial, Medicaid). Comparisons used proportional hazards regression to determine whether prolactin testing was disproportionate with risperidone, regardless of prior potentially prolactin-related adverse events (PPAEs). Logistic regression determined whether magnetic resonance imaging (MRI)/computed tomography (CT) were disproportionate in risperidone-treated patients vs other patients, regardless of hyperprolactinemia or PPAEs. In each regression, the 'other typical' antipsychotic category served as the comparator. Regression models controlled for age, gender, and other factors.

**Results:**

Altogether, 197,926 treatment episodes were analyzed (63,878 risperidone). Among patients with or without preceding PPAEs, risperidone treatment was associated with a significantly greater likelihood of prolactin assessment (hazard ratio (HR) 1.34, 95% confidence interval (CI) = 1.09 to 1.66, p = 0.007). Among patients with hyperprolactinemia or PPAEs, those treated with risperidone (odds ratio (OR) 1.66, 95% CI 1.23 to 2.23, p = 0.001) or ziprasidone (OR 1.66, 95% CI 1.06 to 2.62, p = 0.028) had a higher likelihood of MRI/CT.

**Conclusion:**

Risperidone-treated patients are more likely to undergo prolactin assessment regardless of prior PPAEs, and more likely to undergo MRI/CT in association with hyperprolactinemia or PPAEs. Thus, a predisposition for more evaluations in risperidone-treated patients may contribute to disproportionate identification and reporting of prevalent pituitary adenoma.

## Background

Hyperprolactinemia is a laboratory abnormality that may result from clinical factors such as polycystic ovary disease, thoracic surgery or trauma, or pituitary tumor. Hyperprolactinemia may also be induced by medications, including antipsychotics [1]. The association between antipsychotic medications and hyperprolactinemia has been under investigation since at least the 1970s [2]. Because the release of prolactin from the pituitary gland is inhibited by dopamine, any process resulting in a reduction in dopamine increases prolactin levels [3]. Therefore, antipsychotics, which are believed to exert their therapeutic effect by dopamine receptor blockade, cause prolactin elevation due to loss of inhibition of pituitary lactotrophs [4]. Conventional antipsychotics and the atypical antipsychotic risperidone have been found to raise prolactin levels [2,4-6]. In contrast, other atypical antipsychotics, such as clozapine, quetiapine and olanzapine, have shown smaller or transient effects on serum prolactin levels, possibly because their actions at other receptor sites result in relatively less dopamine blockade [2,4-6], or because of a lower peripheral to central distribution [7].

A 2006 pharmacovigilance study by Szarfman *et al. *found spontaneous reporting of pituitary tumors to be disproportionately higher among patients treated with risperidone compared with other antipsychotics. Based on adjusted reporting ratios (that is, reports of specific adverse events as a proportion of all reports of adverse events for a given medication), reports of pituitary tumors were 8-fold higher in risperidone-treated patients than in olanzapine-treated patients, 31-fold higher than in quetiapine-treated patients, 6-fold higher than in ziprasidone-treated patients, and 3-fold higher than in haloperidol-treated patients. Szarfman *et al*. interpreted these findings as suggesting that risperidone may have a causal relationship with pituitary adenoma [8].

Whereas a potential link between risperidone and pituitary tumor cannot be discounted, there may be other explanations for the considerably higher number of tumors reported with risperidone relative to other antipsychotics. Indeed, further examination of the putative link between risperidone and pituitary tumor is warranted so that clinicians may make informed decisions for their patients, as it may not be practical or desirable to change to another antipsychotic, particularly when the original medication is effective.

Prolactin elevation is a definite concern of antipsychotic treatment, and it is important that prolactin levels be appropriately monitored. Consensus recommendations propose that prolactin levels should be measured if signs and symptoms, elicited through a careful and thorough patient history, suggest hyperprolactinemia. If prolactin levels are elevated in the presence of potentially prolactin-related adverse events (PPAEs) the cause of hyperprolactinemia should be determined, and consideration should be given to changing to a prolactin-sparing antipsychotic [9]. However, clinicians may test prolactin levels routinely in patients who take antipsychotics known to increase prolactin, even in the absence of PPAEs.

Disproportionate prolactin testing in risperidone-treated patients can ultimately lead to the identification of pituitary tumors that would otherwise remain undetected, because such tumors are usually small, benign, and endocrinologically silent [10]. In fact, they generally are discovered only incidentally via brain imaging studies or upon autopsy. A recent meta-analysis found the estimated prevalence of asymptomatic pituitary tumors in the general population to be quite high: 14.4% in autopsy studies and 22.5% in radiological studies [11]. Given that they are quite common, but usually asymptomatic, pituitary lesions found in patients receiving risperidone may be misinterpreted as having an etiologic relationship with the treatment drug.

Szarfman *et al. *used a pharmacovigilance database to examine cases of pituitary tumor. The frequency of diagnosed pituitary tumors can also be determined from claims data. However, claims data may be subject to certain biases. Not all adverse events require or receive medical attention, and the proportion of events that is actually diagnosed may vary across medications. Further, two forms of potential bias may occur in association with risperidone treatment: (1) patients may be more likely to undergo testing for prolactin elevation, regardless of the prior presence of PPAEs, leading to a diagnosis of hyperprolactinemia that otherwise may have remained clinically silent; and (2) risperidone-treated patients, particularly those with PPAEs, may be more likely to undergo investigation that could result in an incidental diagnosis of benign pituitary tumors. Both sources of bias would contribute to a higher frequency of diagnosed pituitary tumors, the first by expanding the patient base and the second, directly. In this context, using claims data, we examined whether there was potential bias in the reporting of pituitary tumors among patients treated with risperidone. Given the relatively high frequency of asymptomatic pituitary tumors in the general population, the effect of these potential biases on the rate of diagnosed pituitary tumors would be potentially large.

## Methods

This study was based on merged claims data from 135,472 patients with either commercial insurance or on public assistance covering the period from 1999 to March 2003 (public assistance) and August 2003 (commercial). Commercial claims were drawn from the PharMetrics patient-centric database and public assistance claims were from the Ohio Medicaid program. All patients with a mental disorder (as per International Classification of Diseases, Ninth Revision, Clinical Modification (ICD-9-CM) codes 290.xx to 316.xx) with at least two sequential prescriptions for the same antipsychotic were included. Comparisons were made among risperidone, clozapine, olanzapine, quetiapine, ziprasidone, haloperidol, perphenazine (each coded individually), and all other typical antipsychotics grouped into a single category. Among the typicals, haloperidol and perphenazine were given individual attention because of their prominence in clinical and other prospective trials.

The sampling unit, which served as the basis for determining frequencies of pituitary tumor, hyperprolactinemia and other related conditions, and diagnostic tests, was the antipsychotic treatment episode (exposure interval) rather than the patient. An antipsychotic treatment episode was defined as a sequence of two or more prescriptions for a specific antipsychotic agent (a subsequent prescription provides reasonable assurance that the first prescription was used). Episodes were measured from the date of the first prescription for an antipsychotic to the final date of treatment with that antipsychotic. The final date was calculated from the date of the last prescription available in the database, plus the number of days for which it was supplied, unless preceded by patient disenrollment from the health plan or the end of the data period, in which case the episode was censored. The first prescription in an episode was based on a prior gap in prescriptions for the defining antipsychotic in excess of 90 days. Gaps of less than 90 days within treatment episodes were allowed. Gaps rarely exceeded 90 days without complete discontinuation of a medication. Some patients had multiple treatment episodes with the same or a different antipsychotic. Additionally, treatment episodes with different antipsychotics overlapped in many cases; thus, a given period for a patient could be characterized by two concurrent exposures. The real-world practice of switching antipsychotics or discontinuing antipsychotic treatment renders use of the patient as the sampling unit inaccurate for associating antipsychotic side effects. Such a treatment episode approach has been used in other published studies [12,13].

To be included, treatment episodes also had to be associated with a prior patient history of at least 180 days. This prior patient history was used to assess prior antipsychotic treatment and pre-existence of pituitary tumor, hyperprolactinemia and PPAEs (ICD-9 diagnostic codes for gynecomastia, galactorrhea, oligomenorrhea, amenorrhea, dysmenorrhea, hypogonadism, hypothyroidism, infertility-male-hypospermatogenesis, infertility-female-pituitary/hypothalamic, impotence-organic, psychosexual dysfunction, genitourinary malfunctions arising from mental factors, and alopecia). Treatment episodes showing pre-existence of any of these prior to the start of treatment with a specific antipsychotic were excluded.

The study focused on the frequency of prolactin tests, head/brain diagnostic procedures, and pituitary tumors diagnosed after the start of each antipsychotic treatment. To avoid false associations, measurement was confined to the treatment episode plus 30 days beyond (unless the episode was censored). This 30-day extension allowed for the inclusion of diagnoses and tests that were triggered by the same circumstances that caused termination of the antipsychotic.

### Investigation bias

Antipsychotics were compared with respect to the likelihood of a patient receiving a prolactin test. Using proportional hazard regression, hazard ratios (HR) were estimated for clozapine, risperidone, olanzapine, quetiapine, ziprasidone, haloperidol, and perphenazine vs all other typical antipsychotics as a single category. The model included factors for type of antipsychotic treatment, prior presence of potentially prolactin-related symptoms (as described above), patient age, gender, concurrent use of antipsychotics, mental disorder diagnoses, and type of insurance. After controlling for the prior presence of symptoms, in the absence of bias, one would not expect to observe any association between the type of antipsychotic treatment and the likelihood of receiving a prolactin test. A significant positive association would reflect a disproportionate tendency to test, irrespective of symptom presentation.

The likelihood of receiving a head/brain magnetic resonance imaging (MRI) or computed tomography (CT) scan was compared among antipsychotic categories. Using logistic regression, odds ratios (ORs) were estimated for clozapine, risperidone, olanzapine, quetiapine, ziprasidone, haloperidol, and perphenazine vs all other typical antipsychotics as a single category. The model included factors for patient age, gender, duration of antipsychotic treatment, presence of hyperprolactinemia or a closely-related condition (gynecomastia, galactorrhea, oligomenorrhea, amenorrhea, and dysmenorrhea), presence of other conditions requiring head/brain imaging studies (skull or brain injury 6 months before or during treatment, skull or brain neoplasm 6 months before or during treatment), concurrent use of antipsychotics, mental disorder diagnoses, type of insurance, and censoring.

A significant interaction between the indicator for hyperprolactinemia or closely-related symptoms and the antipsychotic categories would capture bias in the propensity to screen for pituitary tumors when hyperprolactinemia was present. That is, the interaction tests whether hyperprolactinemia is differentially associated with a diagnostic investigation, depending on the antipsychotic category or, alternatively, whether the association between the diagnostic investigation and antipsychotic category depends on the presence or absence of hyperprolactinemia. Independent associations between the antipsychotics and the use of head/brain imaging studies are of less interest; simply being treated with a particular antipsychotic would not seem sufficient for differential testing for pituitary tumor.

### Reporting bias

Relative frequencies (percentages of treatment episodes) of newly-diagnosed pituitary tumors (as described above) were compared among the antipsychotic categories using ICD-9 coding categories of benign, uncertain, unspecified, and malignant. Because treatment duration (exposure time) varied considerably among the antipsychotic categories, relative frequencies were standardized against a 1-year exposure to adjust for variable treatment episode durations.

## Results

A total of 135,472 patients were identified, receiving a total of 197,926 treatment episodes (exposure intervals) with an antipsychotic medication. The overwhelming majority of these patients had mental disorder diagnoses (ICD-9-CM) of schizophrenia, bipolar disorder, major depression, or dementia. A total of 40,651 patients had multiple treatment episodes (17,235 with the same antipsychotic and 23,416 with a different antipsychotic), averaging 2.54 episodes per patient. The antipsychotics did not differ appreciably with respect to the proportion of patients with multiple episodes. Overall, there were 69,873 episodes with risperidone, 2,093 with clozapine, 56,138 with olanzapine, 36,857 with quetiapine, 7,183 with ziprasidone, 10,743 with haloperidol, 2,956 with perphenazine, and 18,132 with all other typical antipsychotics. There was at least some concurrent use (mostly representing the transition from one antipsychotic to another) in 72,038 of the total 197,926 treatment episodes.

Patient characteristics are summarized in Table 1. Average treatment durations were similar across drugs, except for clozapine and ziprasidone. The longer duration and many other differences were expected in association with clozapine treatment based on its different indicated population (treatment-refractory patients who have failed other options), the requirement for monitoring due to risk of agranulocytosis, use in different settings of care, small exposed population, and other factors. The shorter average treatment duration for ziprasidone was anticipated as a result of its later entry into the market relative to other antipsychotics. Patients treated with typical antipsychotics were generally older than those treated with atypical agents, with ziprasidone-treated patients being the youngest. Gender proportions varied considerably; clozapine was the only agent used in more males than females. Concurrent use of other antipsychotics, particularly other atypical antipsychotics, was relatively low for both risperidone-treated and olanzapine-treated patients.

**Table 1:** Patient characteristics by antipsychotic category

	**Clozapine**	**Risperidone**	**Olanzapine**	**Quetiapine**	**Ziprasidone**	**Haloperidol**	**Perphenazine**	**Other typicals**
Number of treatment episodes	2,039	63,878	56,138	36,857	7,183	10,743	2,956	18,132
Duration of treatment, mean (SD), mo	16.0 (13.8)	10.5 (9.9)	9.9 (9.8)	9.7 (8.9)	7.1 (5.5)	9.8 (9.9)	10.2 (9.7)	9.5 (9.4)
Age, mean (SD), years	45 (16)	44 (25)	46 (21)	41 (20)	36 (16)	53 (21)	52 (19)	50 (18)
Males, %	54.4	46.3	45.3	39.4	41.8	46.5	36.7	40.2
With diagnosis of hyperprolactinemia during treatment, %	0.25	0.44	0.09	0.17	0.25	0.18	0.27	0.25
With prolactin test during treatment, %	1.52	2.06	1.15	1.41	1.84	0.94	1.05	1.08
With potentially prolactin-related symptoms during treatment, %*	9.1	7.1	6.5	7.9	7.9	6.0	7.1	7.0
With head/brain MRI or CT scan during treatment, %	16.8	12.1	11.4	11.9	8.5	13.8	11.6	13.7
With skull/brain injury or neoplasm 6 months prior to or during treatment, %†	4.17	3.94	4.11	3.87	2.28	8.02	2.64	6.48
With diagnosis of non-malignant pituitary tumor during treatment, %								
Benign	0.15	0.13	0.05	0.06	0.11	0.08	0.03	0.07
Uncertain behavior	0.00	0.04	0.01	0.00	0.06	0.00	0.00	0.04
Unspecified	0.05	0.07	0.06	0.05	0.01	0.05	0.00	0.10
Malignant	0.05	0.03	0.01	0.01	0.00	0.02	0.00	0.03
Used another antipsychotic within 6 months prior to treatment, %	71.8	30.2	37.0	50.7	74.7	54.0	46.9	71.8
Concurrent use of other atypical antipsychotic, ratio of days supply to index antipsychotic days supply, mean (SD)	0.32 (0.41)	0.09 (0.25)	0.10 (0.25)	0.16 (0.32)	0.27 (0.39)	0.43 (0.45)	0.30 (0.42)	0.27 (0.40)
Concurrent use of other typical antipsychotic, ratio of days supply to index antipsychotic days supply, mean (SD)	0.16 (0.32)	0.05 (0.18)	0.07 (0.22)	0.07 (0.23)	0.08 (0.23)	0.04 (0.17)	0.05 (0.19)	0.07 (0.22)
Diagnoses, %:								
Schizophrenia	84.4	24.9	30.3	27.5	42.5	52.2	38.3	38.4
Affective psychoses	46.2	50.7	57.1	63.4	64.1	39.0	52.4	40.2
Other psychoses	43.3	37.2	34.2	30.1	26.7	49.8	38.5	28.9
Other non-psychotic mental disorders	66.8	69.3	69.1	74.2	70.1	61.2	61.5	62.4
Health coverage:								
Medicaid	75.6	64.0	62.4	62.5	55.4	75.0	71.9	73.6
HMO	12.5	20.9	19.8	18.0	20.5	15.4	14.7	14.3
Other health coverage	11.9	15.1	17.8	19.5	24.1	9.6	13.4	12.1

*Gynecomastia, galactorrhea, oligomenorrhea, amenorrhea, dysmenorrhea, hypogonadism, hypothyroidism, infertility-male-hypospermatogenesis, infertility-female-pituitary/hypothalamic, impotence-organic, psychosexual dysfunction, genitourinary malfunctions arising from mental factors, and alopecia.

† Based on following International Classification of Diseases, Ninth Revision, Clinical Modification (ICD-9-CM) codes: 851.xx to 854.xx, 900.82, 900.89 and 900.9 for brain or intracranial injury; 801.xx to 804.xx for skull injury; 191.xx, 198.3, 225.0 to 225.2, 237.5, and 239.6 for brain neoplasm and 170.9, 198.5, 213.9, 238.0 and 239.2 for skull neoplasm.

CT, computed tomography, HMO, health maintenance organization; MRI, magnetic resonance imaging; SD, standard deviation.

The majority of patients (55% to 75%, depending on antipsychotic) were covered by Medicaid. Among privately insured patients, a health maintenance organization (HMO) was generally the most prevalent form of coverage, with preferred provider, point-of-service, and other types making up the remainder.

Although a higher proportion of risperidone-treated patients received a diagnosis of hyperprolactinemia after the start of treatment, the proportion of patients with PPAEs was similar among the antipsychotics even after differences in treatment duration were taken into account. Consistent with the more frequent diagnosis of hyperprolactinemia was the more frequent prolactin testing among risperidone-treated patients. The frequency of prolactin tests in risperidone-treated patients was about two times that in patients treated with olanzapine, haloperidol, or perphenazine and about 50% higher than that in patients treated with quetiapine.

Proportional hazards regression results for prolactin tests are reported in Table 2. Among the antipsychotics, risperidone alone was associated with a significantly greater likelihood (HR 1.34, p = 0.007) of prolactin testing compared with the reference group, after controlling for prior presence of potentially prolactin-related symptoms and other patient characteristics. The estimated HR suggests that the likelihood of testing with risperidone was nearly 35% higher than the 'all other typicals' category. Although not statistically significant, estimated HRs for clozapine, olanzapine, quetiapine, haloperidol and perphenazine were all less than 1.0. Prior claims for prolactin-related symptoms, as would be expected, had a large significant effect on the likelihood of prolactin testing (HR 6.74, p < 0.0001). The interaction of risperidone with this variable was also positive and significant (HR 1.41, p = 0.0269), suggesting a 41% greater likelihood of prolactin testing among risperidone-treated patients with PPAEs compared with similarly symptomatic patients treated with 'other typicals'. Interaction terms for the other antipsychotics were not statistically significant.

**Table 2:** Likelihood of receiving a prolactin test: proportional hazards regression results

	**Hazard ratio**	**95% CI**	**p Value**
Antipsychotic categories vs other typicals (excluded category):			
Risperidone (yes = 1)	1.341	1.085 to 1.658	0.0067
Clozapine (yes = 1)	0.748	0.411 to 1.362	0.3418
Olanzapine (yes = 1)	0.948	0.788 to 1.229	0.8894
Quetiapine (yes = 1)	0.927	0.738 to 1.164	0.5124
Ziprasidone (yes = 1)	1.162	0.857 to 1.576	0.3343
Haloperidol (yes = 1)	0.852	0.609 to 1.319	0.3523
Perphenazine (yes = 1)	0.741	0.416 to 1.319	0.3077
Prolactin-related symptoms prior to event or censoring (yes = 1)*	6.736	5.080 to 8.932	< 0.0001
Interaction of antipsychotic and PPAE:			
Risperidone × PPAEs	1.406	1.040 to 1.901	0.0269
Clozapine × PPAEs	1.340	0.617 to 2.911	0.4594
Olanzapine × PPAEs	1.156	0.838 to 1.593	0.3771
Quetiapine × PPAEs	0.936	0.673 to 1.302	0.6929
Ziprasidone × PPAEs	1.068	0.686 to 1.663	0.7710
Haloperidol × PPAEs	1.094	0.676 to 1.770	0.7134
Perphenazine × PPAEs	1.608	0.747 to 3.463	0.3077
Age	0.962	0.959 to 0.964	< 0.0001
Male gender	0.329	0.300 to 0.361	< 0.0001
Used another antipsychotic within 6 months prior to treatment (yes = 1)	1.070	0.984 to 1.164	0.1130
Concurrent use of other atypical antipsychotic, ratio of days supply to index antipsychotic days supply	1.647	1.465 to 1.852	< 0.0001
Concurrent use of other typical antipsychotic, ratio of days supply to index antipsychotic days supply	1.136	0.954 to 1.354	0.1531
Diagnosis:			
Schizophrenia (yes = 1)	1.009	0.925 to 1.101	0.8352
Affective psychosis (yes = 1)	1.318	1.212 to 1.434	< 0.0001
Other psychosis (yes = 1)	0.953	0.879 to 1.032	0.2370
Other non-psychotic mental disorder (yes = 1)	1.266	1.143 to 1.402	< 0.0001
Health coverage vs fee-for-service (excluded category):			
Medicaid (yes = 1)	0.924	0.830 to 1.028	0.1474
HMO (yes = 1)	1.146	1.016 to 1.293	0.0266

Number of observations with event: 2,796; number of observations censored: 195,130.

*Gynecomastia, galactorrhea, oligomenorrhea, amenorrhea, dysmenorrhea, hypogonadism, hypothyroidism, infertility-male-hypospermatogenesis, infertility-female-pituitary/hypothalamic, impotence-organic, psychosexual dysfunction, genitourinary malfunctions arising from mental factors, and alopecia.

CI, confidence interval; HMO, health maintenance organization; PPAEs, potentially prolactin-related adverse events.

Among the other variables in the model, increasing patient age and male gender showed significant decreased associations with the likelihood of prolactin testing. Concurrent use of atypical antipsychotics, diagnoses of affective psychoses and non-psychotic mental disorders, and HMO coverage all showed significant increased associations.

Table 3 shows logistic regression results for head/brain MRI or CT scan. All of the specified antipsychotics, except clozapine, were associated with a significantly lower likelihood of a head/brain diagnostic procedure vs 'other typical' antipsychotics (the excluded category) after controlling for the presence of hyperprolactinemia or a closely-related condition and several other patient characteristics. The presence of hyperprolactinemia or a closely-related condition increased the likelihood of a head/brain diagnostic procedure by nearly 80% (OR 1.78, p < 0.0001). Among patients with hyperprolactinemia or a closely-related condition, those treated with risperidone or ziprasidone were 65% more likely to have undergone a head/brain MRI or CT scan (OR 1.66, p = 0.0009, and OR 1.66, p = 0.0278, respectively) than patients treated with 'other typical' antipsychotics. Clozapine, olanzapine, quetiapine, haloperidol, and perphenazine showed no significant differences from the 'other typicals' group. None of the antipsychotics had independent positive associations with the likelihood of undergoing a head/brain diagnostic procedure.

**Table 3:** Likelihood of undergoing a head/brain MRI or CT scan: logistic regression results*

	**Odds ratio**	**95% CI**	**p Value**
Antipsychotic categories vs other typicals (excluded category):			
Risperidone (yes = 1)	0.812	0.770 to 0.855	< 0.0001
Clozapine (yes = 1)	0.976	0.853 to 1.116	0.7222
Olanzapine (yes = 1)	0.778	0.738 to 0.821	< 0.0001
Quetiapine (yes = 1)	0.901	0.851 to 0.954	0.0003
Ziprasidone (yes = 1)	0.828	0.750 to 0.913	0.0002
Haloperidol (yes = 1)	0.768	0.713 to 0.826	< 0.0001
Perphenazine (yes = 1)	0.709	0.626 to 0.803	< 0.0001
Hyperprolactinemia (inclusive of closely-related conditions*) during treatment (yes = 1)	1.781	1.354 to 2.343	< 0.0001
Interaction of antipsychotic and hyperprolactinemia:			
Risperidone × hyperprolactinemia	1.658	1.232 to 2.232	0.0009
Clozapine × hyperprolactinemia	0.572	0.243 to 1.343	0.1993
Olanzapine × hyperprolactinemia	0.974	0.701 to 1.352	0.8748
Quetiapine × hyperprolactinemia	1.261	0.912 to 1.744	0.1613
Ziprasidone × hyperprolactinemia	1.663	1.057 to 2.615	0.0278
Haloperidol × hyperprolactinemia	1.157	0.741 to 1.805	0.5213
Perphenazine × hyperprolactinemia	1.237	0.568 to 2.696	0.5925
Duration of antipsychotic treatment episode, months	1.034	1.032 to 1.035	< 0.0001
Censored treatment episode (yes = 1)	0.746	0.723 to 0.770	< 0.0001
Age	1.018	1.017 to 1.018	< 0.0001
Male gender (yes = 1)	0.965	0.936 to 0.995	0.0217
Skull or brain injury 6 months prior to or during treatment (yes = 1)	4.956	4.672 to 5.258	< 0.0001
Skull neoplasm 6 months prior to or during treatment (yes = 1)	2.097	1.842 to 2.388	< 0.0001
Brain neoplasm 6 months prior to or during treatment (yes = 1)	8.630	7.687 to 9.689	< 0.0001
Used another antipsychotic within 6 months prior to treatment (yes = 1)	0.944	0.913 to 0.975	0.0005
Concurrent use of other atypical antipsychotic, ratio of days supply to index antipsychotic days supply	1.120	1.068 to 1.174	< 0.0001
Concurrent use of other typical antipsychotic, ratio of days supply to index antipsychotic days supply	1.145	1.075 to 1.220	< 0.0001
Diagnosis:			
Schizophrenia (yes = 1)	1.023	0.989 to 1.057	0.1845
Affective psychoses (yes = 1)	1.429	1.386 to 1.474	< 0.0001
Other psychoses (yes = 1)	2.110	2.047 to 2.174	< 0.0001
Other non-psychotic mental disorders (yes = 1)	1.822	1.758 to 1.888	< 0.0001
Health coverage vs fee for service (excluded category):			
Medicaid (yes = 1)	1.476	1.406 to 1.550	< 0.0001
HMO (yes = 1)	1.084	1.024 to 1.148	0.0053

Number of observations with event: 25,343; number of observations without event: 172,583.

*Conditions closely related to hyperprolactinemia include gynecomastia, galactorrhea, oligomenorrhea, amenorrhea, and dysmenorrhea.

CI, confidence interval; CT, computed tomography; HMO, health maintenance organization; MRI, magnetic resonance imaging.

Among the other variables in the model, as would be expected, longer antipsychotic treatment duration (observation) was associated with a greater likelihood of receiving a head/brain diagnostic procedure, whereas censoring of the treatment episode due to lack of subsequent patient records was associated with a lower likelihood. The presence of a skull/brain injury or neoplasm greatly increased the likelihood of these procedures. Other variables with significant increased associations were increasing patient age, concurrent use of antipsychotics, diagnoses other than schizophrenia, and Medicaid and HMO forms of coverage. Variables with significantly decreased associations included male gender and switch from another antipsychotic.

Consistent with receiving more frequent head/brain diagnostic procedures, diagnosed pituitary tumors, particularly the benign and uncertain behavior types, were also more frequent among risperidone-treated patients (Table 4). Pituitary tumor frequencies were combined across types and adjusted for differences in antipsychotic treatment duration (Table 4). For each antipsychotic category, the frequency was standardized against a 1-year exposure to adjust for variable treatment episode durations. Adjusted frequencies of pituitary tumor in patients treated with clozapine, olanzapine, quetiapine, or haloperidol were very similar to each other, and generally lower than the frequency in risperidone-treated patients. The frequency of claims for pituitary tumors with risperidone was 1.6 to 1.9 times higher than the frequencies with the previously mentioned antipsychotics. The frequency of pituitary tumor among perphenazine-treated patients was by far the lowest; the rate for risperidone-treated patients was eight times higher than that for perphenazine-treated patients. However, frequencies of pituitary tumor in patients treated with ziprasidone or other typical antipsychotics were similar to the frequency in risperidone-treated patients.

**Table 4:** Frequencies of pituitary tumor according to antipsychotic treatment

	Clozapine	Risperidone	Olanzapine	Quetiapine	Ziprasidone	Haloperidol	Perphenazine	Other typicals
Number of treatment episodes	2,039	63,878	56,138	36,857	7,183	10,743	2,956	18,132
Pituitary tumor (all types), %:								
Unadjusted*	0.25	0.26	0.13	0.13	0.18	0.15	0.03	0.24
Adjusted for antipsychotic treatment duration†	0.19	0.30	0.16	0.16	0.30	0.18	0.04	0.30

*Because of rounding, these percentages may differ from the sum of percentages in Table 1.

† Unadjusted percentages were raised or lowered to reflect 12-month treatment duration.

## Discussion

The typical antipsychotics and risperidone have long been known to be associated with a greater propensity to elevate prolactin levels. A recent pharmacovigilance study by Szarfman *et al. *[8] showed a considerably higher proportion of pituitary tumor spontaneous reports in patients treated with risperidone vs patients treated with other antipsychotics, and the authors suggested that this observation may reflect a causal association with risperidone treatment. This explanation is, of course, of great concern, and warrants careful medical review of individual case reports.

We analyzed claims databases to further examine the reported association between risperidone and pituitary tumor because it is generally recognized that pharmacovigilance data do not provide reliable 'denominators' that appropriately characterize the size of the sample at risk. Denominators in pharmacovigilance disproportionality analyses are numbers of adverse events, not numbers of patients or treatment episodes. Thus, adverse event frequencies in pharmacovigilance data may reflect a disproportionate relationship between reported and diagnosed events across medications [14]. Claims data, in contrast to pharmacovigilance data, provide reliable denominators for better ascertainment of the frequencies of adverse events and procedures across agents.

Further, we hypothesized that the reporting association may have been influenced by several sources of reasonably anticipated bias. Widespread awareness of the greater propensity of risperidone to elevate prolactin may lead clinicians to routinely perform tests for hyperprolactinemia (even in patients without attributable symptoms), and subsequently to disproportionately order diagnostic procedures that revealed a coincidental pituitary tumor or false positive related to other causes of pituitary hypertrophy and/or sellar masses (such as craniopharyngiomas, Rathke's cleft cyst, lymphocytic hypophysitis and pituitary enlargement or physiologic hyperplasia) [15-17].

Results of our study indeed suggest that clinicians are more likely to test prolactin levels in risperidone-treated patients, resulting in more hyperprolactinemia diagnoses and a larger pool of candidates for pituitary tumor investigation. Even after controlling for the prior presence of PPAEs, risperidone-treated patients were found to have a significantly greater likelihood (34% more likely) of receiving a prolactin test than patients treated with typical antipsychotics other than haloperidol and perphenazine. Estimates for patients treated with all of the other antipsychotics, except ziprasidone, showed non-significant but lower likelihoods of prolactin testing. Unfortunately, although claims data provide information on whether a prolactin test is performed, they do not provide results of those tests, and so the degree of prolactin elevation is not known.

Importantly, the relative frequency of PPAEs among risperidone-treated patients was similar to that among patients treated with other antipsychotics, even though the rate of diagnosed hyperprolactinemia was higher. These data are consistent with those observed in retrospective analyses [18] and controlled clinical studies of risperidone vs olanzapine [19] and risperidone vs quetiapine [20], which found that although most risperidone-treated patients have some prolactin elevation, clinical effects are uncommon.

Among patients with hyperprolactinemia or a closely-related condition, those treated with risperidone were 65% more likely to undergo a head/brain MRI or CT scan than patients treated with typical antipsychotics other than haloperidol or perphenazine. A similar result was observed for ziprasidone. In contrast, patients in this group treated with clozapine, olanzapine, quetiapine, haloperidol, or perphenazine showed no significant difference in the likelihood of undergoing a head/brain diagnostic procedure.

Although this claims-based study found higher rates of diagnosed pituitary tumor in risperidone-treated patients compared with those treated with most other antipsychotics, demonstrating sensitivity to detection of the 'signal' previously reported, this relative increase was not universally true; risperidone had slightly lower rates than ziprasidone and typical antipsychotics other than haloperidol and perphenazine. Pharmacovigilance data [8], based on disproportionality ratios of spontaneously reported diagnoses of pituitary tumor, found higher rates for risperidone vs other agents that ranged from 3-fold higher (vs haloperidol) to 21-fold higher (vs clozapine). In contrast, in this population-based claims data, ratios of diagnosed pituitary tumor for risperidone vs other antipsychotics ranged from 0.9 (vs ziprasidone) to 1.9 (vs quetiapine). Signal scores reported from the pharmacovigilance data, which are used to detect a potential safety concern, cannot be directly compared to analyses that use patient-based or treatment episode-based denominators.

One limitation of this study, as noted above, is the absence of detailed patient-level clinical information, including prolactin values. As a result, hyperprolactinemia was treated as a categorical measure (yes/no), and we could not establish how the degree of hyperprolactinemia could have impacted the likelihood of head/brain imaging. However, even after controlling for PPAEs, more risperidone-treated patients were tested for prolactin elevation, which is the first, necessary step in the decision pathway leading to diagnostic imaging and subsequent detection of pituitary tumor. Further, it is very likely that PPAEs are underreported in all patients who receive antipsychotics, owing to patient and clinician reluctance to discuss such matters and a greater priority on treating symptoms of mental illness itself.

Although we attempted to control for a variety of available patient characteristics, other characteristics potentially affecting results were impossible to gauge. For example, in many instances, potentially prolactin-related symptoms may not have been reported on medical claims, particularly if they were first noted immediately prior to a prolactin test and diagnosis of hyperprolactinemia. Although such symptoms were almost certainly reported in patient medical records, they would not necessarily be listed on medical claims. To the extent that these omissions were disproportionately likely to occur in risperidone-treated patients, our findings of investigation bias may have been affected. Further, because of the very low frequency of pituitary tumor, we made the decision to include in the study all antipsychotic-treated patients who met data requirements. Patients with a diagnosis of dementia were in this group and accounted for less than 5% of the total, which is not surprising given that the Medicaid and commercially insured populations studied are overwhelmingly non-elderly. However, MRI is often used to assess dementia. This could have affected our findings of differential likelihoods of head/brain diagnostic procedures among the various antipsychotics to the extent that the antipsychotics differed substantially in their proportions of dementia patients and related MRI procedures.

In all, 30% of patients (40,651) had multiple treatment episodes, raising the possibility of interdependence of sampling units. This was assessed and noted to make no difference in the data. Treatment episodes for the same patient were usually separated by long intervals, during which patient circumstances, including health state, may have changed considerably. Additionally, interdependence of sampling units can arise from other factors, such as two patients being treated by the same physician or having the same specific type of health coverage. Meaningful interdependence was addressed in these analyses by the exclusion of observations with evidence of pre-existing hyperprolactinemia, potentially prolactin-related symptoms and pituitary tumor. Therefore, we did not further exclude data or make any adjustments.

## Conclusion

Findings from this large claims-based study, involving nearly 200,000 observations from diverse patient populations, indicate that the disproportional reporting of pituitary tumor in patients treated with risperidone from pharmacovigilance data sets may be influenced by several reporting biases. Although this and other studies cannot establish absence or presence of a causal relationship between atypical antipsychotic treatment generally (and risperidone treatment specifically), and pituitary tumors, it is important to recognize that pituitary tumors of clinical relevance may still occur in patients receiving antipsychotic medication, and that patients with symptoms suggesting pituitary tumor should receive full appropriate evaluation.

## Abbreviations

CT: computed tomography; HMO: health maintenance organization; HR: hazard ratio; ICD: International Classification of Diseases; MRI: magnetic resonance imaging; PPAE: potentially prolactin-related adverse event.

## Competing interests

FG and RW are employees of HECON associates, Inc., a contract research organization. They worked under a contract with Janssen, and have no other affiliations, financial or otherwise, to report. GP is employed by Johnson & Johnson Pharmaceutical Research and Development; RM and JW are employees of Ortho-McNeil Janssen Scientific Affairs, L.L.C.

## Authors' contributions

FG made the following contributions to the manuscript: concept/design, data analysis/interpretation, statistics, data collection, and project administration. GP and RM provided concept/design and data analysis/interpretation. JW provided data analysis/interpretation. RHW provided data acquisition and organization, data analysis/interpretation, and statistics. All authors read and approved the final manuscript.
